# Dr. Eugene B. Horton

**Published:** 1912-06

**Authors:** 


Dr. Eugene B. Horton, City Bacteriologist of Niagara Falls, died May 6, 1912, at the Buffalo General Hospital, of cerebro-spinal meningitis, aged 39. He was a graduate of Buffalo, 1902.

